# Reference ranges for ultrasonographic renal dimensions as functions of age and body indices: A retrospective observational study in Taiwan

**DOI:** 10.1371/journal.pone.0224785

**Published:** 2019-11-07

**Authors:** Hsuan-An Su, Han-Ying Hsieh, Chien-Te Lee, Shang-Chih Liao, Chi-Hsiang Chu, Chien-Hsing Wu

**Affiliations:** 1 Department of Medical Education, Kaohsiung Chang Gung Memorial Hospital, Kaohsiung, Taiwan; 2 Department of Pediatrics, Tung’s MetroHarbor Hospital, Taichung, Taiwan; 3 Division of Nephrology, Department of Internal Medicine, Kaohsiung Chang Gung Memorial Hospital, Kaohsiung, Taiwan; 4 Clinical Trial Center, Kaohsiung Chang Gung Memorial Hospital, Kaohsiung, Taiwan; Istituto Di Ricerche Farmacologiche Mario Negri, ITALY

## Abstract

An ethnicity-based reference range for normal renal size is fundamental for ultrasonographic assessment. Herein, we aimed to establish a Chinese renal reference by a large sample size, as well as to elucidate the relationship of renal dimension to age and body indices, with the aid of a comprehensive literature review. Records of 3707 healthy cases were obtained from health evaluation centers of Kaohsiung and Linkuo Chang Gung Memorial Hospitals. As a result, the mean right renal length was 10.62±0.69 cm, left renal length 10.76±0.70 cm, right renal width 4.78±0.75 cm, and left renal width 5.10±0.64 cm. Renal size was well-correlated curvilinearly to age, while linearly to body height, body weight, and body mass index. Renal size increases and then decreases with aging, and significant variations of renal size exist among different ethnicities. Our work provides a reliable reference range for renal size in the Chinese population, and valid relationships between renal dimensions and other parameters.

## Introduction

Ultrasonography is one of the most useful techniques for evaluating kidney morphology [1]. Its ease of accessibility, non-invasiveness, cost effectiveness, and safety make it the most commonly used modality to study renal morphometry [2].

Renal size is of great clinical importance for screening, diagnosis, and follow-up of renal diseases, as the basis of clinical decisions. Serial measurements are helpful in determining disease progression or stability [3]. Decreased renal size usually indicates a chronic change, while increased renal size implies an acute change, diabetic condition, or other structural abnormalities [4–7]. Currently, kidney references published by Dinkel et al. in 1985 [8], Rosenbaum et al. in 1984 [9], Miletić et al. in 1998 [10], or other studies based on Caucasian populations, widely serve as reference for patients in Taiwan and other countries [2, 11, 12]. However, the sample sizes included were limited, the years of publication were outdated, and the populations were mainly Caucasian, which could lead to misjudgment in clinical practice. The establishment of an appropriate and valid reference of normal Taiwanese renal size is fundamental, but has been omitted for decades.

Renal morphology has been reported to be correlated to body indices, such as age, sex, body height (BH), body weight (BW), and body mass index (BMI) [2, 13], with discrepant results reported by studies from different populations. The correlation between renal morphology and body indices allows for the evaluation of renal health conditions by quick estimation, and in forensic medicine, it allows for the identification of individuals beyond exterior recognition [13]. Inconsistent reports on the correlations between renal morphology and body indices exist in the literature. For example, some researchers reported negative correlations between renal size and age [2, 10, 12, 14–17], but some researchers found no correlations [13, 18–22]. Since the roles of body indices in reflecting renal size are controversial, an analysis with a large sample size is required.

To the best of our knowledge, a comprehensive study on a Chinese ultrasonographic renal reference with a very large sample size has not been previously reported, with the largest sample size so far of 4035 reported in a Pakistani study in 2011 [15]. In the current retrospective study, we aimed to establish a reference range for normal renal dimensions in a healthy Taiwanese adult population using a large sample size of 3707 over a recent eight-year period, and to elucidate the correlations between renal sonographic findings and body indices.

## Materials and methods

### Renal sonography in healthy subjects

Ultrasonographic renal measurements from January 4, 2010, to December 29, 2017, were retrospectively obtained from renal sonographic examinations performed in Chang Gung Memorial Hospital (CGMH) and Healthcare Centers of Linkou and Kaohsiung branches in Taiwan. The examinees underwent abdominal ultrasonography as a part of self-paid general health examinations. The extraction protocol was approved by the Institutional Review Board of Chang Gung Medical Foundation (IRB No. 201802374B0). Extracted data included examination date, sonographic examiner, chart number, renal length, renal width, cortical thickness of both sides in centimeters, and other abnormal findings. Abnormal sonographic findings were also extracted, which were diagnosed according to the examiners’ judgements and experiences. Data on age, sex, BH, BW, BMI, and underlying morbidities such as hypertension, diabetes mellitus, or chronic kidney disease (CKD) were also obtained from the medical profiles. Exclusion criteria were HbA1c ≥6.5 mg/dL; serum creatinine level >1.27 mg/dL in males and >1.02 mg/dL in females; estimated glomerular filtration rate (eGFR) <60 mL/min/1.73 m^2^ in the Modification of Diet in Renal Disease study equation (MDRD); medical history of hypertension, diabetes mellitus, or any renal disease; and abnormal ultrasonographic findings that may influence renal size, including renal cyst, hydronephrosis, renal stone, pelviectasis, polycystic kidney disease, acute change (e.g. acute pyelonephritis), status post-nephrectomy, renal mass, adrenal mass, chronic progressive nephropathy, gouty nephropathy, medullary calcinosis, columnar hypertrophy, renal duplication, renal abscess, and diabetic nephropathy. Although BMI >24 indicates overweight or obesity, individuals with BMI≥24 were not excluded from our analysis, because the Taiwanese population is one of the most obese populations in Asia, and not excluding these overweight or obese individuals reflects the genuine situation of our population [23].

Real-time gray-scale ultrasound was performed with a 3.5 MHz curvilinear probe (Model: PVT-378BT) and TOSHIBA SSA-700: Aplio and TOSHIBA SSA-680: Xario ultrasound machine. Sonography was performed with patients in the prone position with empty urinary bladders. Acoustic gel was applied to the skin to obliterate the air interface between the probe and skin. The gray-scale amplification gain and time-gain compensation curve were adjusted to acquire the best images of the kidneys. Focus number was automatically adjusted with this ultrasound unit, but one focus mode was preferred. Focus was adjusted at the level of the kidney. Tissue harmonic imaging was routinely used. The kidney was clearly identified as having a brightly echogenic renal capsule with a central echogenicity. All examinations were performed by 80 well-experienced nephrologists based on the same measuring protocol. The superior and inferior poles were clearly identified and marked in the longitudinal scan of the kidney, and the renal length was taken as the longest distance between the poles using an electronic caliper. The renal width was measured on a transverse scan, and the maximum transverse diameter at the hilum was taken as the renal width. The renal cortical thickness was defined as the maximal length from the cortex-perirenal fat interface to the cortex-pyramidal base interface measured perpendicularly under longitudinal ultrasound image. The unit of measurement was centimeter (cm).

### Statistical analysis

The extracted data were organized using Microsoft Excel software and analyzed by Statistical Package for Social Sciences (SPSS) Statistics version 22. Descriptive statistics, Student’s t-test, linear regression analysis, and Pearson’s correlation coefficient were used for elaborating demographic and crude estimates, for evaluating differences between continuous variables, for analyzing linear relationships between renal measurements and age or body indices, and for elucidating the correlations between variables, respectively. In univariate linear regression analysis, independent variables included age, the square of age, BH, BW, and BMI, while dependent variables included measurements of renal dimensions, including renal length, renal width, and renal cortical thickness. Variables for modeling the renal dimensions were further identified by multivariate linear regression model with stepwise selection. Analysis of bivariate and partial correlation was performed to examine the confounding effects among age, BH, and BW on right renal length (RL). Statistical significance was considered at *p*<0.05.

### Review of the literature

To compare the variability among different populations, a brief review of the literature on the ultrasound-measured renal lengths in healthy populations has been conducted. PubMed, Scopus, and Airiti Library were searched for relevant studies reported in English or Chinese, without limitation of the date of publication. From the collected studies, countries, right renal length (RL), sample size, age, BW, and BH were extracted as mean and S.D. For studies with insufficient information, data were deduced from the provided data or otherwise left blank. The RLs of different populations were compared with independent Student’s t-test, except for those incomparable data composed of subjects with distinct age distribution.

## Results

Initially, renal sonographic data of 35,554 cases from Kaohsiung CGMH and 222,231 cases from Linkuo CGMH were considered. After exclusion of invalid, unqualified, and diseased data, 3707 independent measurements were included in the final analysis. Among the 3707 measurements included, 1431 (38.60%) were from females and 2276 (61.40%) were from males. Age, BH, BW, and BMI were 49.07±10.77 years (range, 19–85), 166.54±8.51 cm (range, 137.8–192.1), 66.38±12.73 kg (range, 33.0–129.3), and 23.70±3.46 kg/m^2^ (range, 13.43–43.90), respectively. Table 1 shows the basic data of male and female subjects, with measurements for males significantly higher than those for females.

**Table 1:** Basic data of male and female subjects.

	Male	Female	*p* value
N	2276 (61.40%)	1431 (38.60%)	
Age (year)	48.60±10.81	49.82±10.65	<0.001*
Body Height (cm)	171.37±6.07	158.86±5.74	<0.001*
Body Weight (kg)	72.37±10.91	56.86±9.09	<0.001*
BMI (kg/m^2^)	24.51±3.27	22.41±3.37	<0.001*

The data are shown as mean ± S.D.

*Significant results computed with independent-samples t-test.

The ultrasonographic renal measurements of the subjects are listed in Table 2. The mean RL was 10.62±0.69 cm; right renal width (RW), 4.78±0.75 cm; right cortical thickness (RCT), 1.46±0.32 cm; left renal length (LL), 10.76±0.70 cm; left renal width (LW), 5.10±0.64 cm; and left cortical thickness (LCT), 1.49±0.30 cm. All ultrasonographic renal measurements were significantly higher in men than in women (*p*<0.001).

**Table 2:** Ultrasonographic renal measurements of male and female subjects.

Parameters	All	Male	Female
RL (cm)	10.62±0.69	10.76±0.66* (N = 2275)	10.41±0.67* (N = 1430)
RW (cm)	4.78±0.75	4.99±0.73* (N = 1463)	4.46±0.66* (N = 942)
RCT (cm)	1.46±0.32	1.51±0.31* (N = 1392)	1.39±0.31* (N = 901)
LL (cm)	10.76±0.70	10.87±0.69* (N = 2276)	10.59±0.68* (N = 1431)
LW (cm)	5.10±0.64	5.29±0.60* (N = 1464)	4.82±0.59* (N = 942)
LCT (cm)	1.49±0.30	1.52±0.29* (N = 1392)	1.45±0.29* (N = 901)

Data shown as mean ± S.D. Abbreviations: RL, right renal length; RW, right renal width; RCT, right cortical thickness; LL, left renal length; LW, left renal width; LCT, left cortical thickness.

*Significant difference between sexes with independent-samples t-test (*p*<0.001)

**Fig 1:**
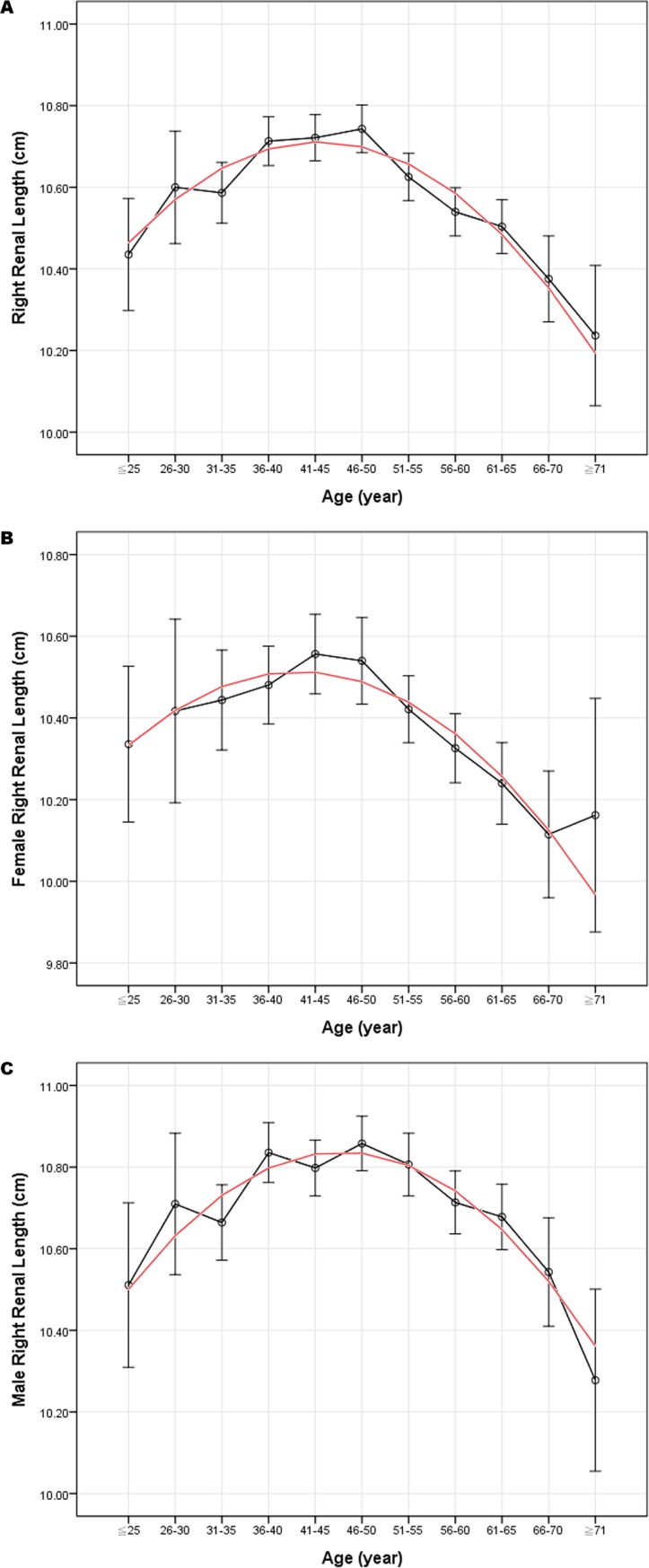
Correlation between age and right renal length (RL). (A) The correlation between age and RL in both sexes is presented as a curvilinear line with a downward opening, and the renal length decreases at the fourth decade of life. The error bars indicate 95% confidence intervals (*p*<0.001). (B) The correlation between age and RL in females is also presented as a curvilinear line with a downward opening. The error bars indicate 95% confidence intervals (*p*<0.001). (C) The correlation between age and RL in males is also presented as a curvilinear line with a downward opening. The error bars indicate 95% confidence intervals (*p*<0.001).

**Fig 2:**
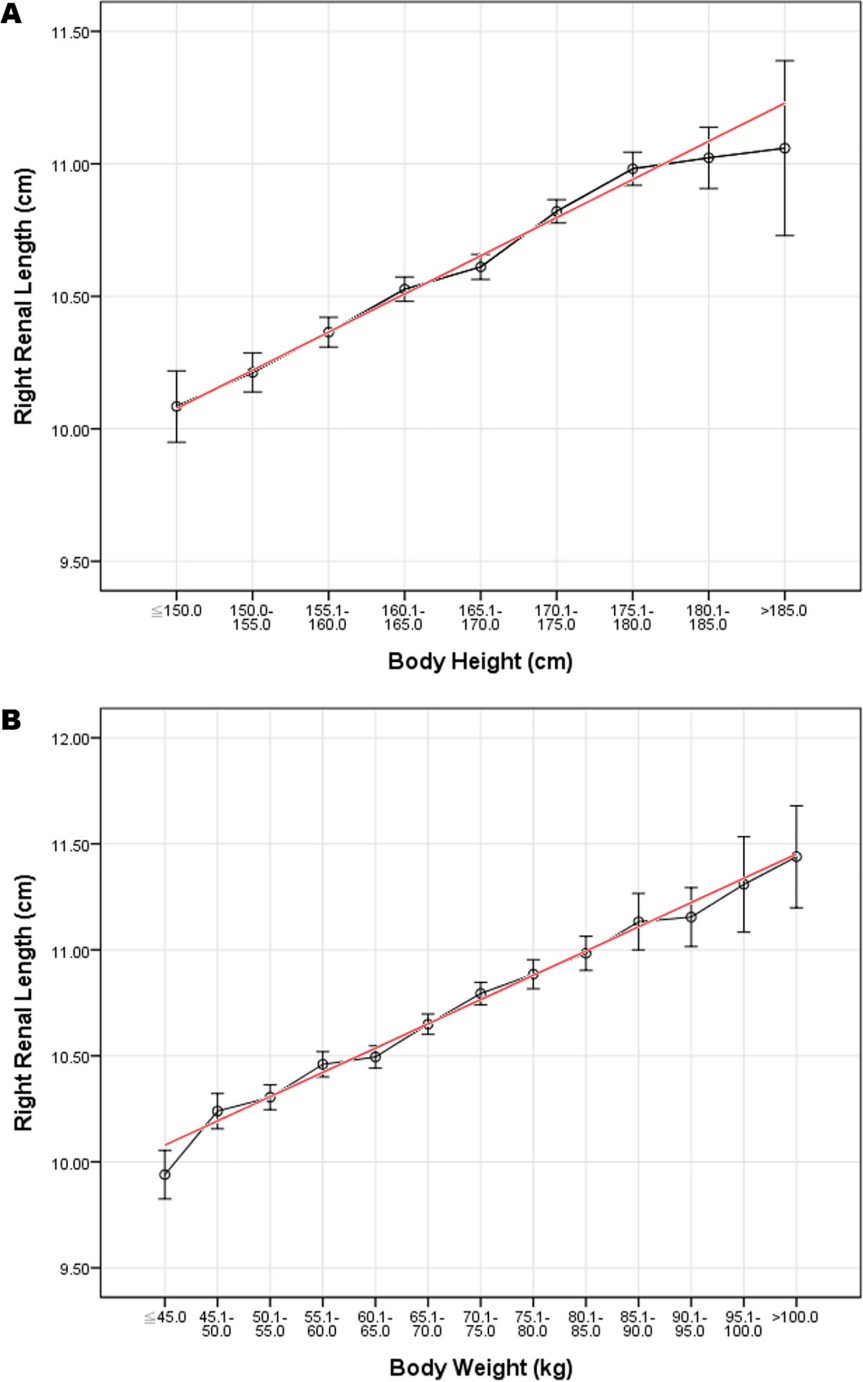
Correlation between body height (BH) or body weight (BW) and right renal length (RL). (A) BH and RL are positively well-correlated. The error bars indicate 95% confidence intervals (*p*<0.001). (B) BW and RL are positively well-correlated. The error bars indicate 95% confidence intervals (*p*<0.001).

Correlation coefficients between renal size and body indices were calculated as shown in Table 3. Among the parameters, BW correlated best while age correlated least; however, all *p*-values indicated significant correlations. By a simple linear regression analysis, the best-fitting lines were illustrated over the distribution graphs of renal dimensions with 95% confidence interval bars of the prediction value, in correspondence with age, BH, BW, and BMI (Figs 1A and 2, Figures A-U in S1 Fig). As age increased, renal length and renal width first increased to the maximum approximately around the fourth decade of life, and then decreased thereafter. Age was quadratically and negatively correlated with renal length and renal width; on the other hand, cortical thickness decreased linearly as age increased. Renal length, width, and cortical thickness were best presented linearly in positive correlations with BH, BW, and BMI. With sex considered separately, the correlations between renal size and body indices were similar with those when calculated together (Fig 1B and 1C, Figures V-AA in S1 Fig).

**Table 3:** Correlations between renal size and age or body indices.

		RL	RW	RCT	LL	LW	LCT
Age^†^	R	-0.155	-0.051	-0.091	-0.133	-0.113	-0.101
	*p* value	<0.001	0.045	<0.001	<0.001	<0.001	<0.001
BH	R	0.363	0.299	0.199	0.329	0.343	0.160
	*p* value	<0.001	<0.001	<0.001	<0.001	<0.001	<0.001
BW	R	0.422	0.399	0.227	0.395	0.429	0.194
	*p* value	<0.001	<0.001	<0.001	<0.001	<0.001	<0.001
BMI	R	0.300	0.322	0.167	0.289	0.333	0.148
	*p* value	<0.001	<0.001	<0.001	<0.001	<0.001	<0.001

^†^The correlation coefficients between age and renal size are obtained by linear regression analysis in which both age and square of age are taken into consideration as variables.

Abbreviations: RL, right renal length; RW, right renal width; RCT, right cortical thickness; LL, left renal length; LW, left renal width; LCT, left cortical thickness.

**Fig 3:**
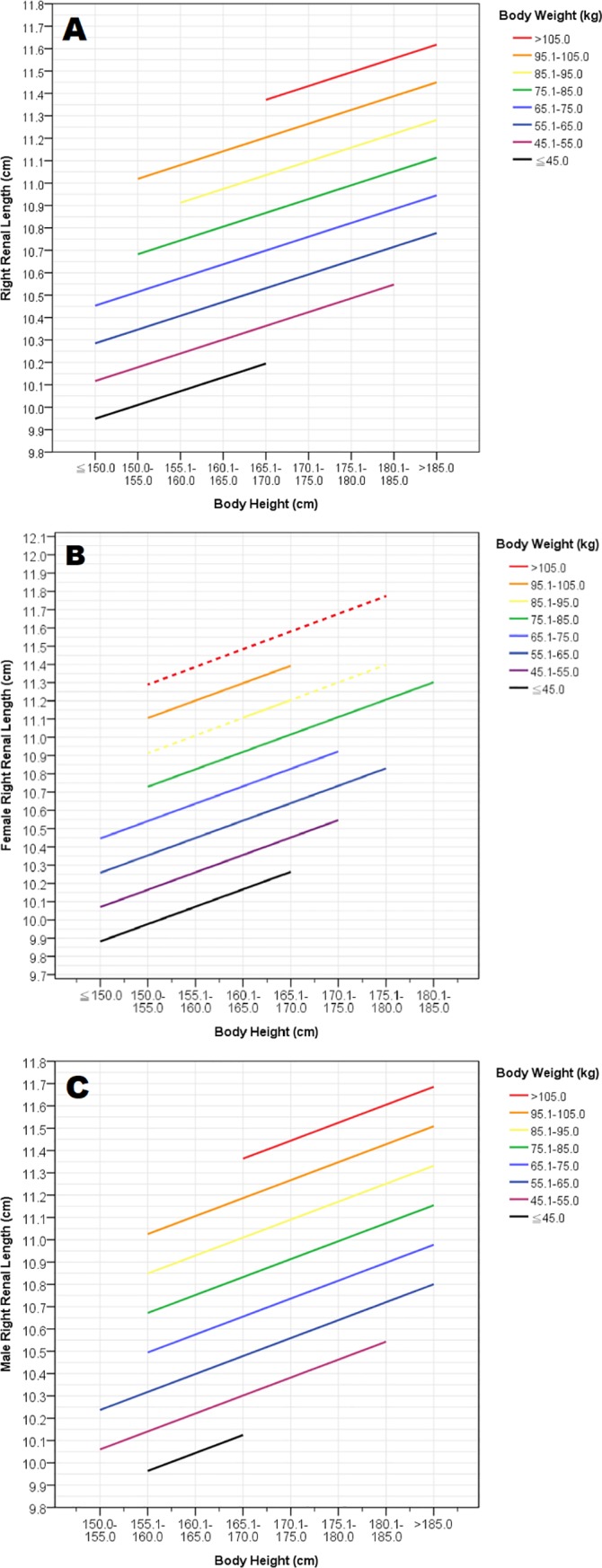
Reference ranges indicating right renal length (RL) by body height (BH) and body weight (BW) of a healthy Taiwanese adult population, with stratification of BW into 8 groups. (A) Reference range of all studied subjects. (B) Reference range of female subjects. (C) Reference range of male subjects. The dotted lines shown in Fig 3B were deduced extensions of the regression lines generated from relatively insufficient data owing to multiple subdivision by sex, BH, and BW.

Based on the aforementioned results, the reference ranges of both sexes for RL was generated as Fig 3. Because age plays a relatively minor role in renal morphology compared to BH and BW, the reference range for RL in the graph illustrated as means of the predicted values, was correlated to BH with stratification of BW.

**Fig 4:**
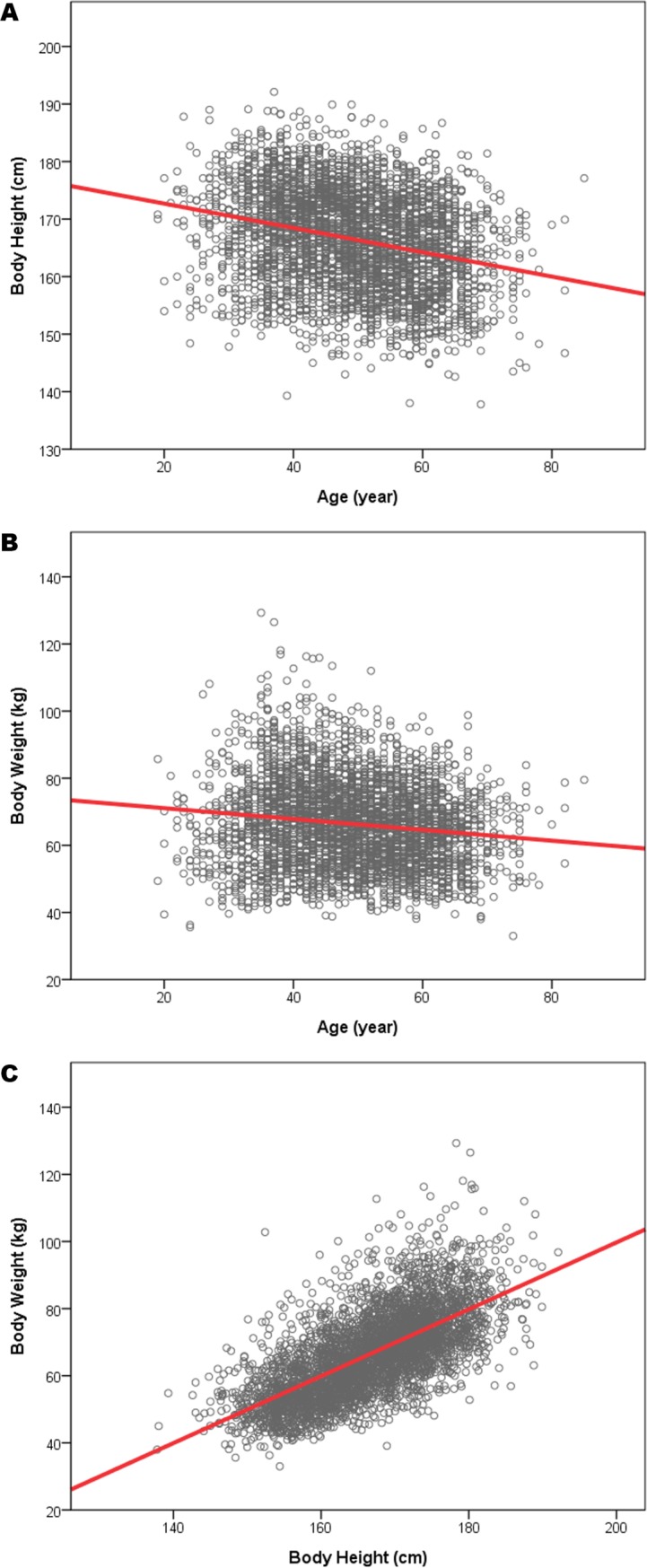
Scatter diagrams with linear regression illustrating the relationships among age, BH, and BW. The linear regression lines demonstrated negative correlations of (A) BH to age (adjusted r^2^ = 0.071, *p*<0.001), (B) BW to age (adjusted r^2^ = 0.018, *p*<0.001), and (C) BW to BH (adjusted r^2^ = 0.444, *p*<0.001).

A stepwise multivariate analysis was performed for the prediction of RL using parameters including age, sex, BH, BW, and BMI (Table 4). RL was presented to represent the results of both sides, and the Pearson’s correlation coefficient between the two sides was 0.663 with statistical significance. The stepwise regression prediction model included square of age, age, sex, BH, BW, and BMI, with the adjusted square of correlation coefficient r^2^ = 0.192. In order to examine the confounding effects of age, BH, and BW upon RL, bivariate correlation analysis revealed that age was negatively well-correlated with both BH (r = -0.267, *p*<0.0001) and BW (r = -0.137, *p*<0.0001). Similarly, age was negatively well-correlated with BH (r = -0.340, *p*<0.001) and BW (r = -0.201, *p*<0.001) in males, while correlated with only BH (r = -0.305, *p*<0.001) but not BW (r = -0.008, *p* = 0.378) in females. By partial correlation analysis, the correlation coefficient of RL to age substantially decreased from -0.102 (*p*<0.001) to -0.006 (*p* = 0.734) after controlling for BH, and also decreased to -0.049 (*p* = 0.003) after controlling for BW. The result of partial correlation analysis showed diminished contribution of age to RL by controlling BH and BW. The relationships among age, BH, and BW were illustrated in scatter diagrams with linear regression lines (Fig 4). The prediction formula demonstrating the correlations among the three variables could be written as follows: *BH* = −0.211*Age*+176.914 (adjusted r^2^ = 0.071, *p*<0.001); *BW* = −0.162*Age*+74.315 (adjusted r^2^ = 0.018, *p*<0.001); and *BW* = 0.997*BH*−99.593(adjusted r^2^ = 0.444, *p*<0.001), with BH, BW, and age in centimeters, kilograms, and years, respectively.

**Table 4:** Univariate and stepwise multivariate regression models for right renal length.

Variables	Univariate model		Multivariate model	
	β (95% C.I.)	*p* value	β (95% C.I.)	*p* value
Age	0.151 (0.100, 0.201)	<0.001		
Square of age	-0.015 (-0.019, -0.011)	<0.001		
Sex^†^	-0.345 (-0.390, -0.301)	<0.001	0.155 (0.095, 0.215)	<0.001
Body height	0.144 (0.132, 0.156)	<0.001	0.083 (0.065, 0.101)	<0.001
Body weight	0.114 (0.106, 0.122)	<0.001	0.095 (0.084, 0.106)	<0.001

^†^Male is substituted by 0, while female is substituted by 1.

The bilateral difference of kidneys was shown with LL, LW, and LCT significantly larger than RL, RW, and RCT, respectively (Figure AB in S1 Figs). The mean difference (±S.D.) was 0.139±0.57 cm (*p*<0.001) for renal length, 0.32±0.67 cm (*p*<0.001) for renal width, and 0.03±0.21 cm (*p*<0.001) for cortical thickness. Similarly, left-sided predominance was also identified in both sexes (data not shown).

**Fig 5:**
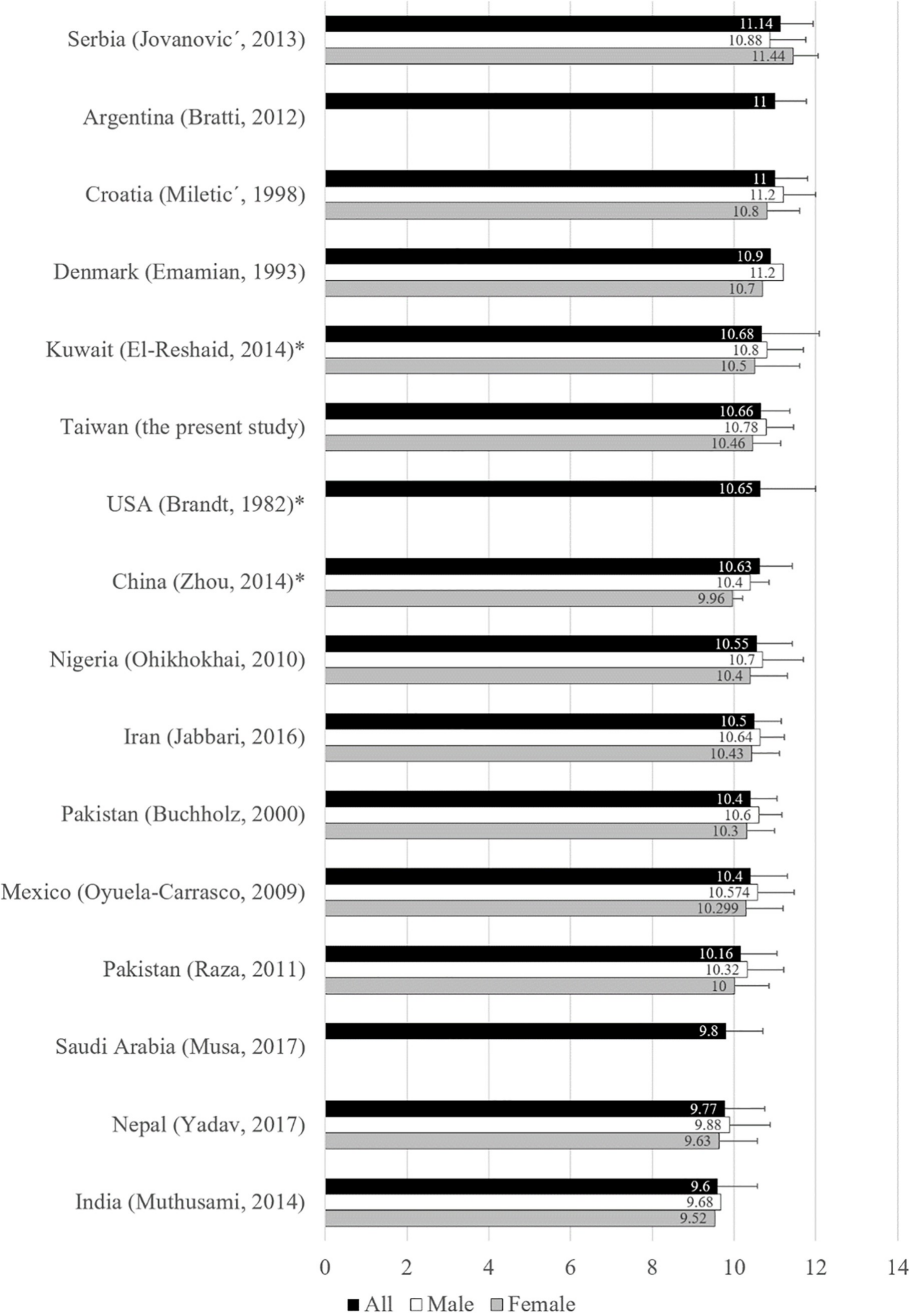
Comparison of right renal length (RL) measured by ultrasonography from different healthy populations with approximately comparable age distribution to the population of the present study. *Data without significant difference compared with our data.

Fifteen studies have been identified that also investigated the renal size of healthy adults using ultrasonography of their own populations with a comparable age distribution to the population in the present study [2, 10, 12–16, 18, 19, 22, 24–28], not including data from pediatric populations, diseased subjects, or non-ultrasonographic measurements. Fig 5 lists the mean RL of various healthy populations measured by ultrasound including the present study, and Table 5 shows the details of the populations. Serbia had the largest mean RL of 11.44±0.80 cm, while India had the shortest mean RL of 9.6 cm. European Caucasian populations—Serbian, Argentinian, Croatian, and Danish—had the top 4 largest RL, respectively. Zhou et al. and our group reported populations from Chinese descendants, and our populations were the eighth (10.63±0.79 cm) and sixth (10.66±0.70 cm) largest in the comparison. Independent samples t-tests revealed significant differences between the RL of our Taiwanese data and those from other studies, except the results of 3 studies by Zhou et al. from China (*p* = 0.2369), Brandt et al. from the USA (*p* = 0.8912), and El-Reshaid et al. from Kuwait (*p* = 0.6846) [18, 24, 28]. On the other hand, other several studies also reported healthy ultrasonographic renal measurements of different populations, yet the age distribution of these selected populations were directly incomparable with the present study, since age is a contributive factor for renal size [11, 17, 20, 21, 29–35].

**Table 5:** Comparison of right renal length (RL) measured by ultrasonography from different healthy populations with approximately comparable age distribution to the population of the present study. Body height and body weight are also shown in the table if available.

Population		RL (cm)	n	Age (year)	BH (cm)	BW (kg)
Serbia (Jovanovic´, 2013)	A	11.14±0.80	46	45.45±18.48 (20–79)	172.25	71.45±12.78
	M	10.88±0.87	20	47.91±19.35	176.15	79.00±9.96
	F	11.44±0.61	26	41.28±15.13	166.75	62.4±9.63
Argentina (Bratti, 2012)	A	11±0.78	98	53.36 (19–85)	-	-
Croatia (Miletic´, 1998)	A	11±0.80	175	46.3±17.1 (17–85)	172.35 (157–192)	-
	M	11.2±0.80	104	-	176±7 (172–192)	-
	F	10.8±0.80	71	-	167±6 (157–185)	-
Denmark (Emamian, 1993)	A	10.9	665	(age groups 30–70)	-	-
	M	11.2	358	-	-	-
	F	10.7	307	-	-	-
Kuwait (El-Reshaid, 2014)	A	10.68±1.40	252	37.73 (18–80)	164.17 (143–190)	76.61(37–124)
	M	10.8±0.90	111	37.9±11.3	172±6.5	85±19
	F	10.5±1.10	141	37.6±13.6	158±6.5	70±14
Taiwan (the present study)	A	10.62±0.69	3707	49.07±10.77 (19–85)	166.54±8.51 (137.8–192.1)	66.38±12.73 (33.0–129.3)
	M	10.76±0.66	2276	48.60±10.81	171.37±6.07	72.37±10.91
	F	10.41±0.67	1431	49.82±10.65	158.86±5.74	56.86±9.09
USA (Brandt, 1982)	A	10.65±1.35	100	(19–62)	-	-
China (Zhou, 2014)	A	10.63±0.79	1000	35.1 (21–78)	166.97	65.82
	M	10.4±0.46	462	35.1±10.1	173.65±6.29	73.95±9.99
	F	9.96±0.25	538	35.1±10.9	161.24±5.64	58.83±7.95
Nigeria (Ohikhokhai, 2010)	A	10.55±0.88	600	39.103 (18–84)	150–190	-
	M	10.7±1.00	309	39.2±14.2	170±10	-
	F	10.4±0.90	291	39±16.2	160±10	-
Iran (Jabbari, 2016)	A	10.5±0.66	103	42.70 (18–70)	164.96 (150–190)	71.79 (43–117)
	M	10.64±0.59	34	40.147±10.4769	174.441±7.4516	77.529±13.7140
	F	10.43±0.68	69	43.957±12.4066	160.290±5.8513	68.957±11.9702
Pakistan (Buchholz, 2000)	A	10.4±0.65	194	44.7±14 (13–80)	-	-
	M	10.6±0.57	98	46.1±15.4 (13–80)	-	-
	F	10.3±0.69	96	43.3±13.2 (15–80)	-	-
Mexico (Oyuela-Carrasco, 2009)	A	10.4±0.90	153	46.12±15.44 (20–79)	160±8.62cm	68.87±11.69
	M	10.574±0.90	77	-	166±6.15 (155–185)	73.77±11.29 (52–111)
	F	10.299±0.90	76	-	154.7±5.97 (139–167)	63.9±9.90 (43.5–85)
Pakistan (Raza, 2011)	A	10.16±0.89	4035	44.4±15.2 (mostly 40–50)	(120–192)	(36–137)
	M	10.32±0.89	1961	-	172.6±6.9	76.3±14.4
	F	10±0.86	2074	-	155.2±5.9	7.1±13.9
Saudi Arabia (Musa, 2017)	A	9.8±0.90	125	(20–70, mostly 41–50)	-	-
Nepal (Yadav, 2017)	A	9.77±0.98	110	35.58±15.45 (15–80)	160±6.75	73.04±12.13
	M	9.88±1.01	57	37.66±17.44	167±6.8	78.45±11.92
	F	9.63±0.94	53	33.33±12.76	153±6.7	67.26±9.44
India (Muthusami, 2014)	A	9.6	140	(18–72, mostly 18–50)	-	-
	M	9.68	69	-	-	-
	F	9.52	71	-	-	-

Data are shown as mean ± S.D. (range). Abbreviations: A, all; M, male; F, female; RL, right renal length; BH, body height; BW, body weight.

## Discussion

In the present study, we ascertained reference ranges for renal size by ultrasonography in a Taiwanese population using a large sample size (Fig 3). Additionally, we also presented significant relationships of renal size to age and body indices. Although this is not a population-based study, the present study includes medical records of an 8-year period with significant results, and Linkuo and Kaohsiung CGMHs are two of the largest and the most famous medical centers in Taiwan, located at the northern and southern Taiwan, respectively. Hence, we believe our results are useful to the Taiwanese population and even to the Chinese population worldwide.

In consistency with our finding, most studies investigating healthy renal size reported negative or insignificant correlations between renal size and age [2, 6, 10, 12, 15, 18, 22, 25, 33], and we would like to further emphasize the quadratic regression with a downward opening, reaching the maximal renal size around the fourth decade of life [16, 22, 32, 34–38]. Piras et al. also investigated the relationships of kidney size to aging and sex in a general population. Although the curves of parenchymal kidney volume with aging were quadratic only in men but not in women, the curves of renal length with aging were quadratic in both sexes [37]. While the results of Piras et al. and ours indicated that renal size starts to decrease at the fourth decade of life, Wang et al. reported decreases of cortical and parenchymal volumes at around the second or the third decade of life [37, 38]. The pathophysiology of decreasing renal size from middle-age onward has been well-discussed [32, 35, 37], including glomerular atrophy within the renal cortex, loss of glomerular lobulation, increase in mesangial volume, glomerulosclerosis, tubulointerstitial fibrosis, reduction in renal blood flow, and intimal hyalinosis of the renal arteries.

As shown in Table 3, although age was significantly correlated with renal size, the correlation was not as strong as other parameters. For example, the correlation coefficient was -0.155 between age and RL, while that between BW and RL reached 0.422. Besides, in stepwise multivariate analysis, age was also excluded from the model, suggesting its negligible influence on RL compared to other parameters. By bivariate correlation analysis, age was negatively correlated with both BH and BW; by partial correlation analysis, the correlation coefficients of RL to age showed substantial decreases after controlling for BH or BW. Thus, the contribution of age to renal size could be partially explained by BH and BW. Therefore, age is one of the contributive but not decisive factors for renal size, and the stature of a person remains the most influential factor.

Despite some studies supporting the role of renal volume in clinical practice because of its better accuracy and correlation with anthropometric parameters [14, 19, 32], renal length has been preferred for routine ultrasonographic measurements, to exclude perplexing calculations and technical inconvenience [25, 31]. Although age was conventionally selected as the independent variable in the graphs of references for renal size [10, 12, 16, 18], BH and BW were chosen as independent variables for renal length in the present study since renal length was much better correlated to BH and BW instead of age. Because of differences in male and female renal morphology, separate reference ranges should be employed for each gender, as illustrated in Fig 3B and 3C.

Every population has the need for its own renal size reference [2, 11–13, 15, 16, 32, 36], which is also supported by the literature review with Fig 5 and Table 5. For example, a RL 12.3 cm of a 45-year-old Taiwanese male would be considered as abnormally oversized beyond 2 S.D. according to results of the present study, but would be considered as normal within 2 S.D. according to the reference published by Miletić et al. in 1998 [10]. The former scenario would lead to an advanced examination for diabetes mellitus or other possible renal diseases, while the latter scenario would neglect a potential patient.

There are conflicting reports in the literature on correlations between renal size and age or body indices. Some authors concluded that renal dimensions were negatively correlated with age in either all or certain age groups [2, 10, 12, 14–17], while some found no correlations between the two [13, 18–22]. Positive correlations between renal width and age have also been reported [19, 25]. Positive correlations between renal length and BH have been reported by most of the studies [2, 10–12, 14–17, 21, 25, 28, 31], but some authors identified no significant correlations [13, 18]. Likewise, most of the studies found positive correlations between renal length and BW [2, 11–13, 15, 16, 18, 21, 31], but Krairittichai et al. found no significant correlation [20], and Jovanović et al. found a significant positive correlation only between renal width and BW [19]. Renal size has been reported to be positively correlated with BMI [2, 11–16, 18, 19, 22, 31, 32, 38], although Krairittichai et al. reported no significant correlation [20]. As for sex differences, men had greater renal sizes than women [10, 12, 14–17, 19–22, 31], although the difference was insignificant in some studies [2, 11, 13, 18]. Except for the studies by Yadav et al. and Muthusami et al. reporting insignificant results [12, 13], most of the studies reported that left renal sizes were significantly larger than right renal sizes [2, 10, 11, 14, 15, 17–19, 21, 22, 25, 31]. The discrepancies among the abovementioned studies could possibly be attributed to the variation in genetic profiles among populations, lack of a sufficient number of cases to reflect the facts, or potentially undiscovered bias on ultrasonographic measurement, definition, or statistical analysis. In any case, renal morphologies differ among populations. The application of renal references based on different populations should be performed with caution and is not recommended.

We acknowledge some limitations of our study. First, it was a retrospective study; hence, some data could be missing and biases were inevitable. Second, ultrasonography-based diagnoses have certain limitations. Some similar abnormal findings were indistinguishable under ultrasonography and the detection of some lesions requires not only expertise but also experience. For instance, a small hyperechoic lesion could be considered as a tiny renal stone or a calcification spot; the former would be excluded from the analysis whilst the latter would not. Third, the sonographic findings were defined qualitatively (e.g. the existence of a renal cyst or hydronephrosis) but not quantitatively (e.g. the diameter of the cyst or the severity of hydronephrosis), which failed to reflect the true effect of the renal abnormalities on renal size. Fourth, to survey intra- and inter-observer variability, analysis of the interclass correlation coefficient (ICC) was technically infeasible because cases in which one case was evaluated by different operators were limited to reflect the true variability among all operators, which was also a disadvantage of the retrospective study design. Instead, one-way ANOVA was conducted for differences in RL measurements, where only 48 high-volume operators who performed ≥ 10 cases in the study were analyzed, and the measurements of RL by the operators were not statistically different (*p* = 0.3451). Because the measurements were assigned to the examiners randomly, though not precise, the results should be considered valid without significant difference. Moreover, due to the established measurement protocol, it is believed that variability among the operators was insufficient to cause notable deviation, and could also be minimized by a great number of cases. Finally, only a few of the contributing factors for renal morphology were identified in the present study, and there were unmeasured confounding factors in the analysis that could also affect renal size, such as waist circumference, underlying diseases not recorded, low birth weight, prematurity, or low nephron numbers [39], etc.

In conclusion, using a large sample size, we propose reference ranges for renal size applicable to the Chinese population. With aging, renal length first increases and then decreases around the fourth decade of life. Renal length, width, and cortical thickness of both sides correlated with age, BH, BW, and BMI, either quadratically or linearly. The inter-racial variation of renal morphology cannot be ignored, and every ethnicity requires a group of reference ranges of its own.

## Supporting information

S1 Figs Correlations between renal dimensions and body indices with 95% confidence interval, and the differences in renal measurements between bilateral kidneys. (DOCX)
