# Supplementary material for: Reference ranges for ultrasonographic renal dimensions as functions of age and body indices: A retrospective observational study in Taiwan
Source: PLoS One. 2019 Nov 7;14(11):e0224785. doi: 10.1371/journal.pone.0224785 (PMC6837751; doi:10.1371/journal.pone.0224785)

Figure A. Correlation between age and RW with 95% confidence interval. (*p*=0.019)


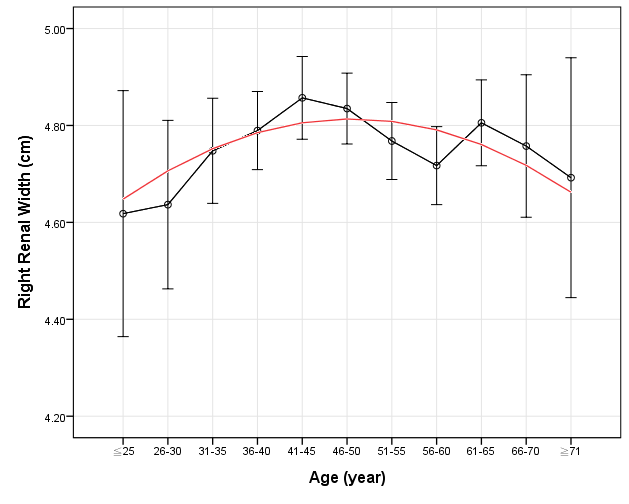


Figure B. Correlation between age and RCT with 95% confidence interval. (*p*<0.001)


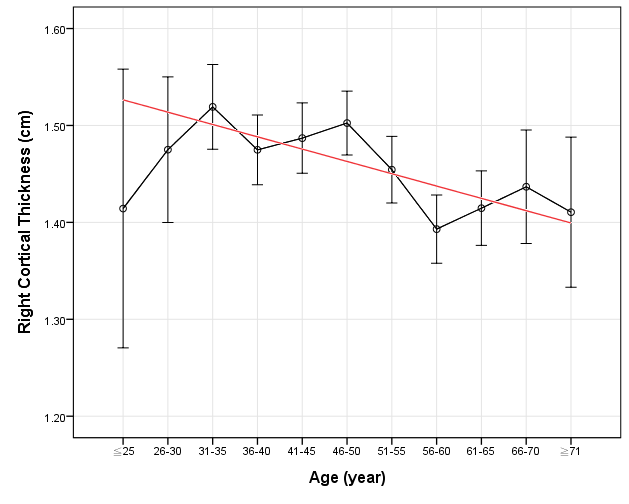


Figure C. Correlation between BH and RW with 95% confidence interval. (*p*<0.001)


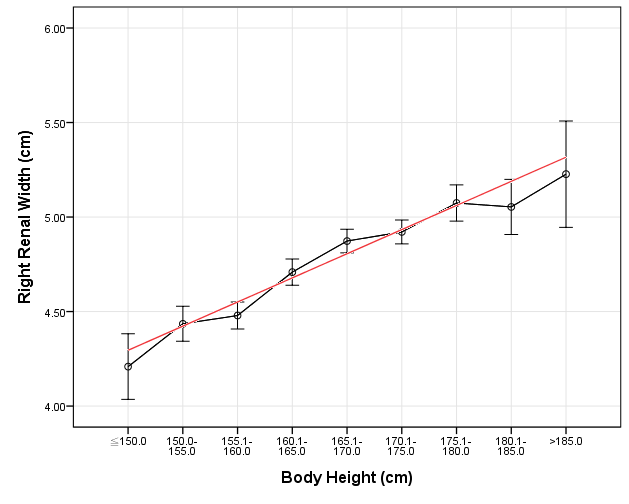


Figure D. Correlation between BH and RCT with 95% confidence interval. (*p*<0.001)


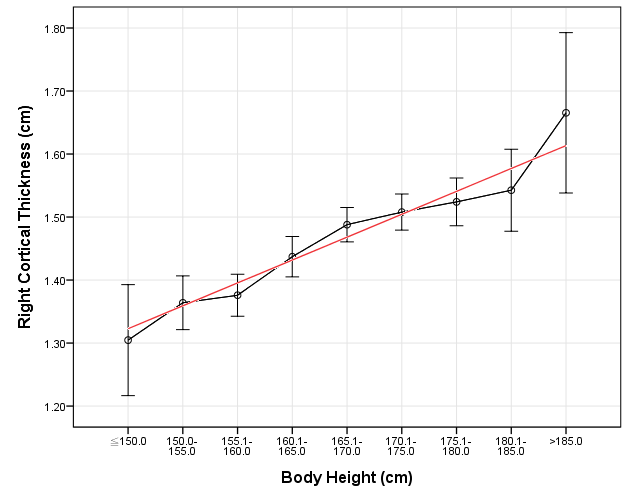


Figure E. Correlation between BW and RW with 95% confidence interval. (*p*<0.001)


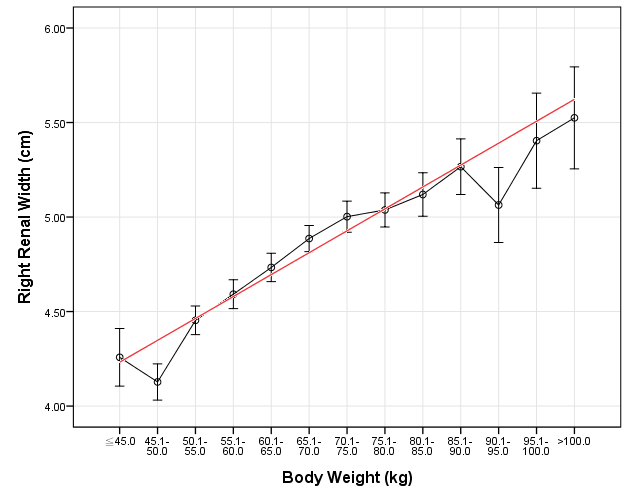


Figure F. Correlation between BW and RCT with 95% confidence interval. (*p*<0.001)


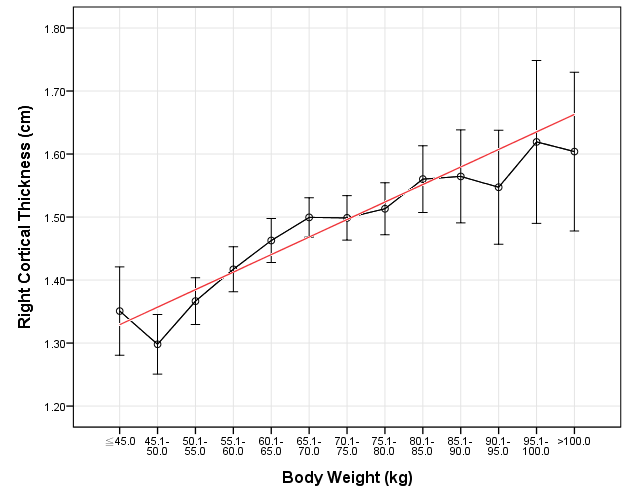


Figure G. Correlation between BMI and RL with 95% confidence interval. (*p*<0.001)


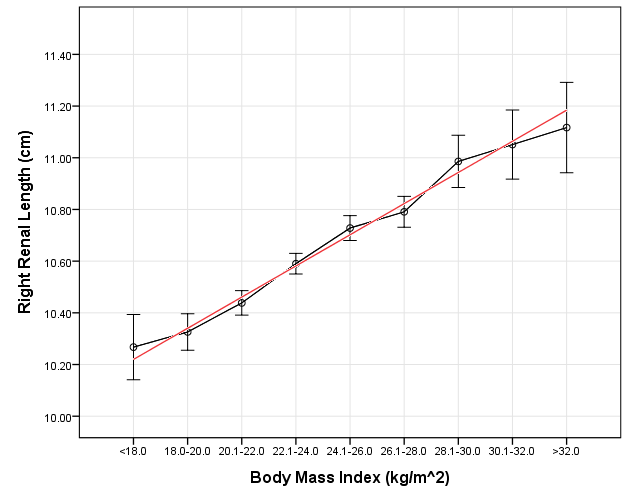


Figure H. Correlation between BMI and RW with 95% confidence interval. (*p*<0.001)


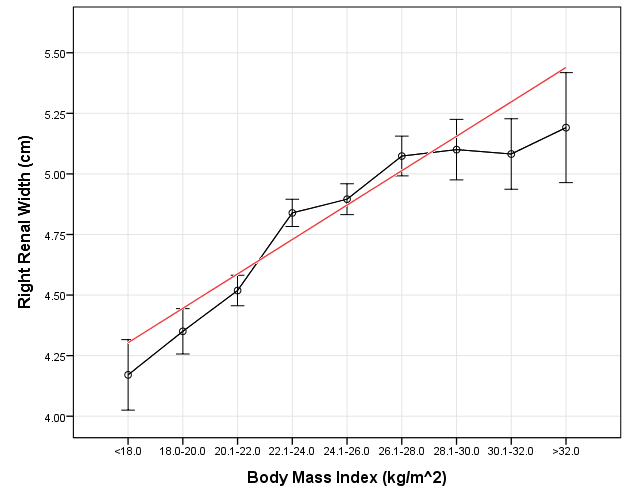


Figure I. Correlation between BMI and RCT with 95% confidence interval. (*p*<0.001)


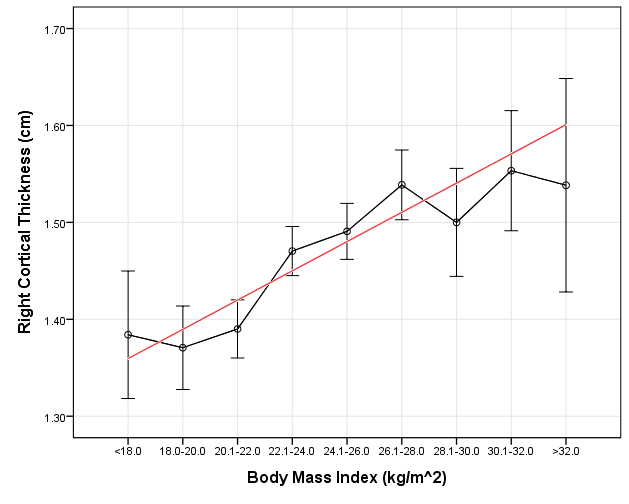


Figure J. Correlation between age and LL with 95% confidence interval. (*p*<0.001)


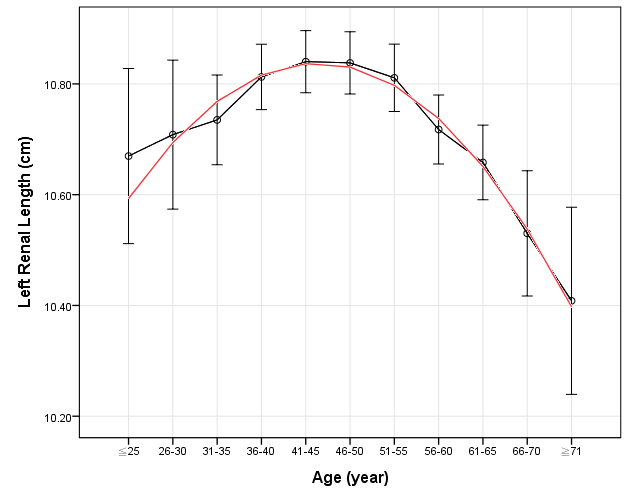


Figure K. Correlation between age and LW with 95% confidence interval. (*p*<0.001)


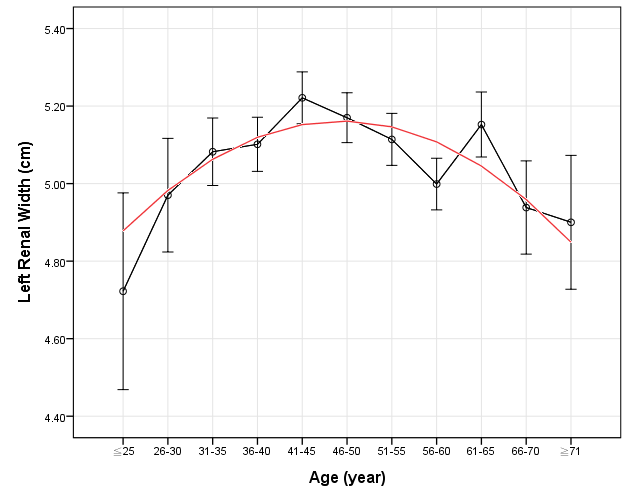


Figure L. Correlation between age and LCT with 95% confidence interval. (*p*<0.001)


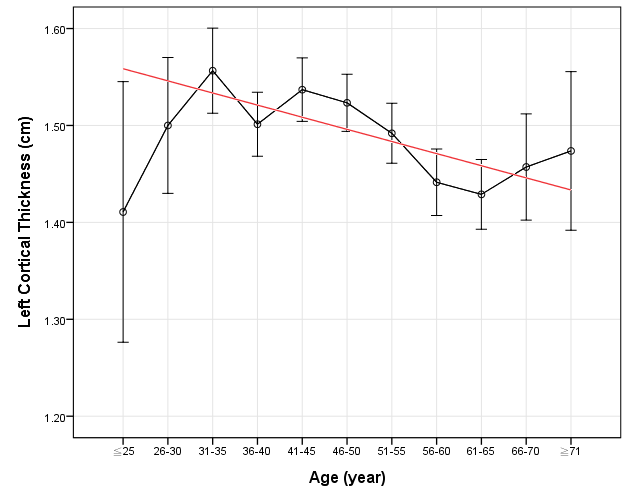


Figure M. Correlation between BH and LL with 95% confidence interval. (*p*<0.001)


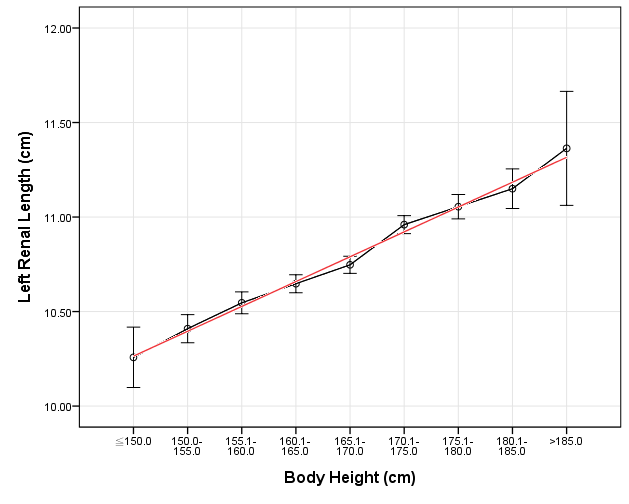


Figure N. Correlation between BH and LW with 95% confidence interval. (*p*<0.001)


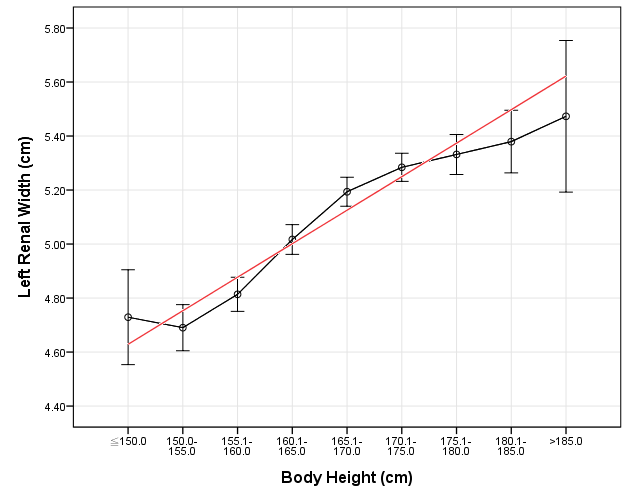


Figure O. Correlation between BH and LCT with 95% confidence interval. (*p*<0.001)


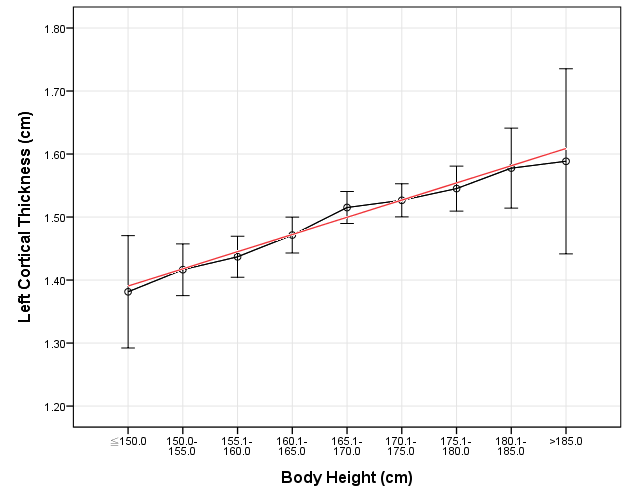


Figure P. Correlation between BW and LL with 95% confidence interval. (*p*<0.001)


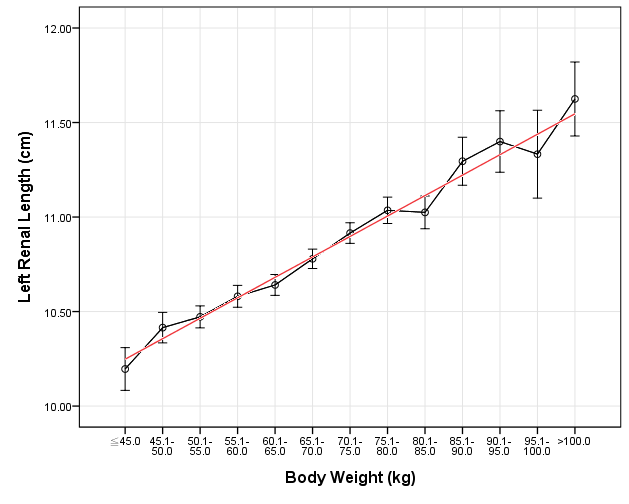


Figure Q. Correlation between BW and LW with 95% confidence interval. (*p*<0.001)


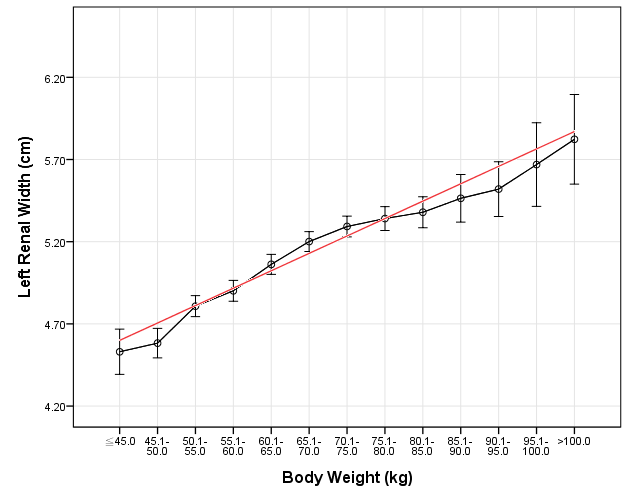


Figure R. Correlation between BW and LCT with 95% confidence interval. (*p*<0.001)


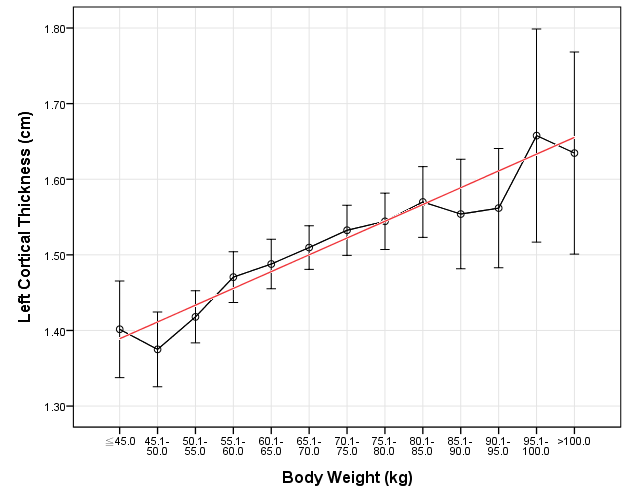


Figure S. Correlation between BMI and LL with 95% confidence interval. (*p*<0.001)


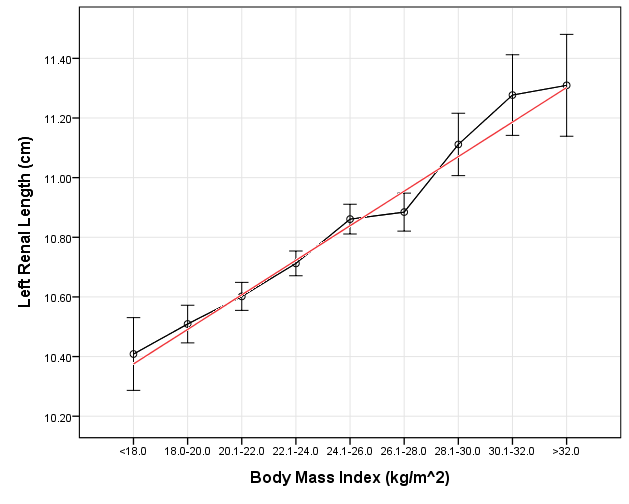


Figure T. Correlation between BMI and LW with 95% confidence interval. (*p*<0.001)


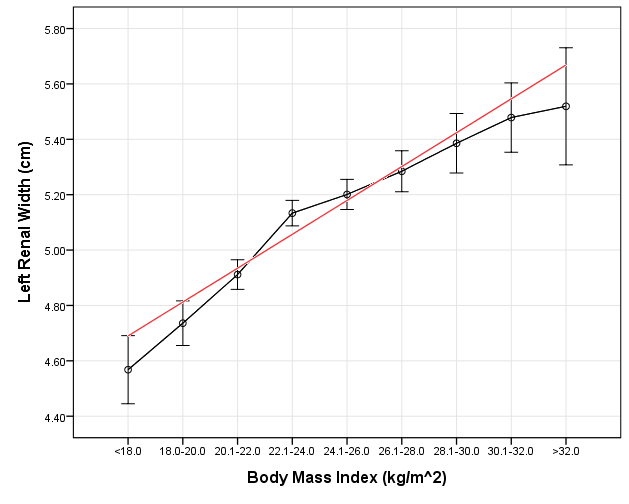


Figure U. Correlation between BMI and LCT with 95% confidence interval. (*p*<0.001)


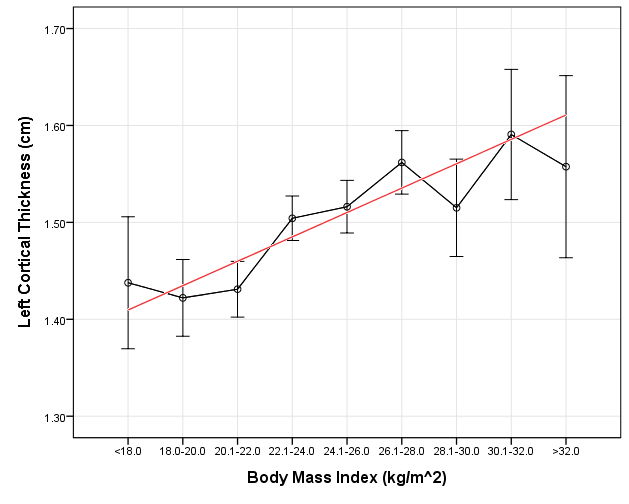


Figure V. Correlation between BH and female RL with 95% confidence interval. (*p*<0.001)


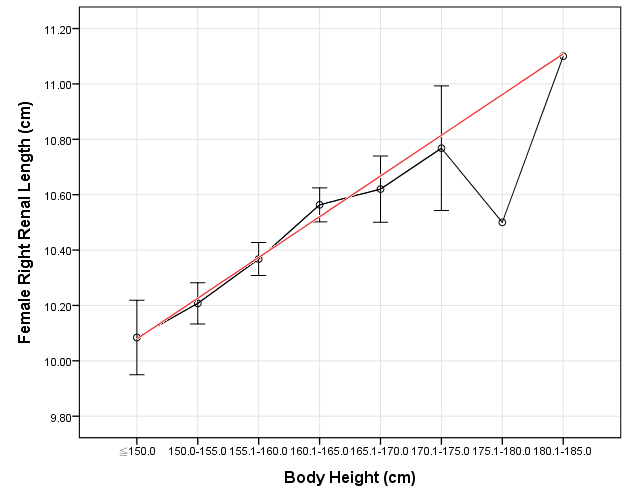


Figure W. Correlation between BH and male RL with 95% confidence interval. (*p*<0.001)


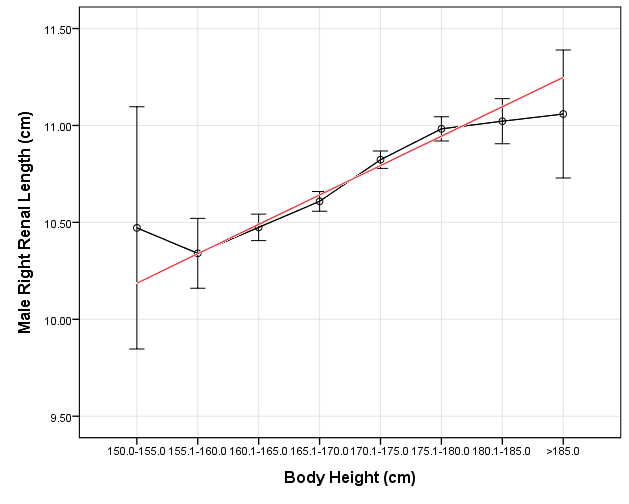


Figure X. Correlation between BW and female RL with 95% confidence interval. (*p*<0.001)


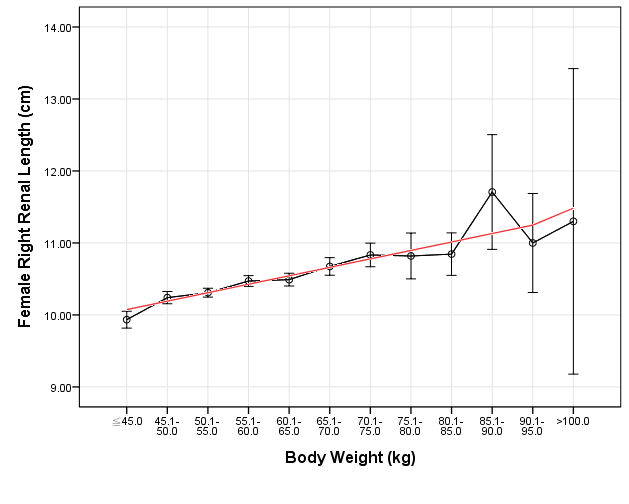


Figure Y. Correlation between BW and male RL with 95% confidence interval. (*p*<0.001)


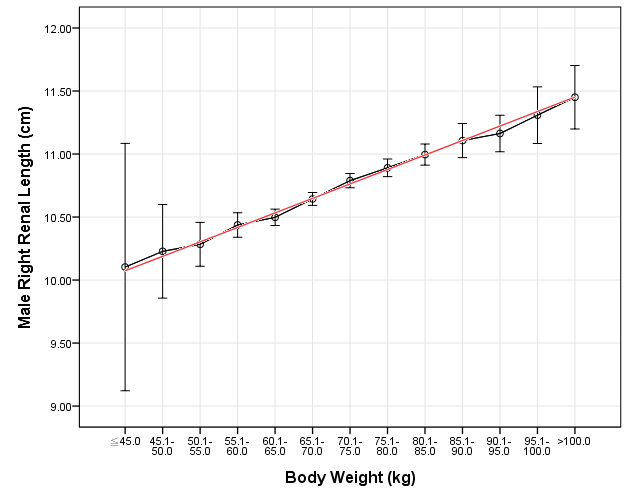


Figure Z. Correlation between BMI and female RL with 95% confidence interval. (*p*<0.001)


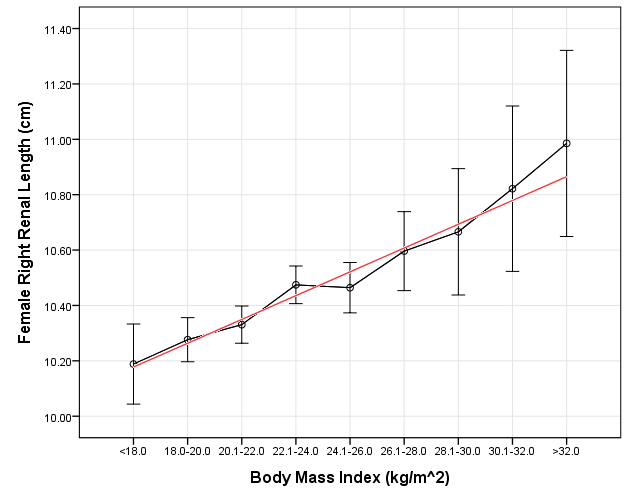


Figure AA. Correlation between BMI and male RL with 95% confidence interval. (*p*<0.001)


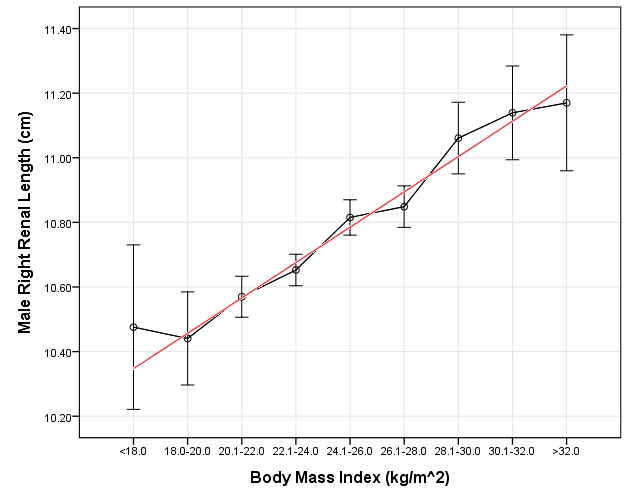


Figure AB. Mean differences with 95% confidence intervals of bilateral kidneys, showing the left significantly larger than the right in length (*p*<0.001), width (*p*<0.001), and cortical thickness (*p*<0.001).


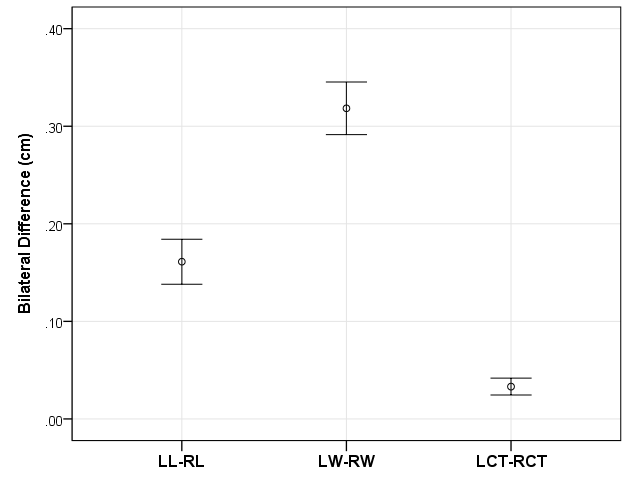

Supplement: S1 Figs — (DOCX) [file pone.0224785.s001.docx]
